# Compton Scattering of γ-Ray Vortex with Laguerre Gaussian Wave Function

**DOI:** 10.1038/s41598-018-37096-3

**Published:** 2019-01-10

**Authors:** Tomoyuki Maruyama, Takehito Hayakawa, Toshitaka Kajino

**Affiliations:** 1 College of Bioresource Sciences, Nihon University, Fujisawa, 252-0880 Japan; 2 National Astronomical Observatory of Japan, 2-21-1 Osawa, Mitaka, Tokyo 181-8588 Japan; 3 National Institutes for Quantum and Radiological Science and Technology, Tokai, Ibaraki 319-1106 Japan; 4 Beihang University, School of Physics, Int. Center for Big-Bang Cosmology and Element Genesis, Beijing, 100083 China; 5 Department of Astronomy, Graduate School of Science, University of Tokyo, Bunkyo-ku, Tokyo 113-0033 Japan

## Abstract

In this work, we report calculation for Compton scattering of a *γ*-ray vortex with a wave function of Laguerre Gaussian on an electron in the framework of the relativistic quantum mechanics. We consider the coincidence measurement of the scattered photon and the scattered electron from each Compton scattering. The momentum of the scattered photon distributes outside of the reaction plane determined by the incident photon and the scattered electron, and the energy of the scattered photon also distributes, when the scattered angle of the electron is simultaneously measured. These distributions depend on the angular momentum and the node number of the Laguerre Gaussian function of the incident photon. Thus, the coincident measurement for Compton scattering is useful to identify the nature of the vortex photon wave function.

## Introduction

Photon vortices with helical wave fronts carrying orbital angular momentum^1^ are interesting both for the fundamental research^2–6^ and for applications^7–15^. For example, it is suggested that photon vortices could be created around rotating black holes^16^. Furthermore, the concept of the vortex has been extended to various beams such as electrons^17,18^ and neutrons^19,20^. One of topics in the photon vortex is the generation of high energy X/*γ*-ray vortices and the interaction with an atomic nucleus or particle. It was demonstrated that X-ray vortex beams were generated by high-order harmonic radiations from helical undulators with high energy electrons^14,15^, and it was presented that a single free electron in circular or spiral motion emits a *l*th harmonic photon carrying *l*ℏ total angular momentum in classical electromagnetism^21^. The energy of the generated photon can be increased by increasing the electron energy, and thus high energy *γ*-ray vortices can be generated. The *γ*-ray vortex generation using inverse Compton scattering with low energy vortex photons on high energy electrons has been studied^22–24^. Furthermore, Taira *et al*.^25^ have proposed the generation of *γ*-ray vortices by nonlinear inverse Compton scattering with a highly intense circularly polarized laser. Thus, it is expected to generate *γ*-ray vortices in the MeV energy region in the near future. However, there is a critical problem that optical devices such as holographic phase plates to measure light vortices at visible wavelengths cannot work in the MeV energy region. Thus, one should invent a new method to verify generated *γ*-ray vortices.

Compton scattering is the dominant process between photons and atoms in the energy range from several hundred keV to several MeV. Because the differential cross section of Compton scattering of linearly polarized *γ*-rays depends on the angle between the scattering plane and the polarization plane, polarimeters based on Compton scattering have been used in nuclear physics^26^ and *γ*-ray astronomy^27^. Thus, Compton scattering is a possible candidate to identify *γ*-ray vortices. In previous studies, Compton scattering with *γ*-ray vortex beams was calculated in non-relativistic framework^6^, and the inverse Compton scattering to generate high energy photon vortices was studied using the relativistic calculation^22,23^.

In this paper, we focus our attention on Compton scattering in order to identify *γ*-ray vortices. We consider the coincident measurement of the scattered photon and electron from each Compton scattering. We calculate the differential cross-section of Compton scattering of *γ*-ray vortices with a wave function of Laguerre Gaussian (LG)^1^ on an electron at rest in the framework of the relativistic quantum mechanics. When a photon with the LG wave function propagates along the *z*-axis, the LG wave function is written as

1 $$u({\bf{r}})=\sqrt{\frac{1}{\pi {R}_{z}}}\frac{1}{w(z)}G[|L|,p,\frac{\sqrt{2}r}{w(z)}]\exp \{i[L\varphi +kz+\frac{z{r}^{2}}{{z}_{R}{w}^{2}(z)}-\mathrm{(2}p+|L|+\mathrm{1)}{\theta }_{G}]\}$$

with

2 $$\begin{array}{c}G[|L|,p,x]=\sqrt{\frac{2p!}{(|L|+p)!}}{x}^{|L|}{e}^{-{x}^{2}/2}{{\mathscr{L}}}_{p}^{|L|}({x}^{2}),\\ {\theta }_{G}={\tan }^{-1}(\frac{z}{{z}_{R}}),\,w(z)={w}_{0}\sqrt{1+{z}^{2}/{z}_{R}^{2}},\,{z}_{R}=k{w}_{0}^{2}/2,\end{array}$$

where *L* is the projection of the orbital angular momentum for the photon propagation axis, *p* is the number of nodes in the transverse direction, ${ {\mathcal L} }_{p}^{|L|}$ is the associated Laguerre function, *k* is the energy of the incident photon, *R*_*Z*_ is the size of the system along the *z*-direction, and *w*_0_ is the waist radius at *z* = 0. The LG wave is described with the two-dimensional harmonic oscillator wave-functions characterized with *L* and *p*. One of the most important properties of the LG wave is that it can have the non-zero *z*-component of the orbital angular momentum, namely *L* ≠ 0. The total angular momentum of the incident photon, which is larger than or equal to (*L* + *s*_*z*_) where *s*_*z*_ is the projection of the spin, is conserved in the scattered photon-electron system. Thus, by measuring simultaneously both the final momenta of the scattered photon and electron, we can investigate the total angular momentum of the incident photon.

## Result

**Figure 1:**
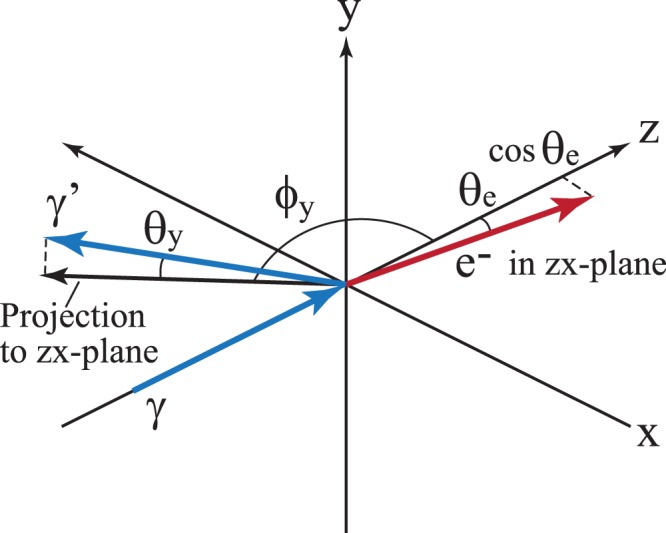
Coordinate system used in the calculation. *γ* and *γ*′ denote the initial and final photons, respectively. The electron as e^−^ is scattered in the *zx*-plane.

When a photon with a LG wave function propagates along the *z*-axis and the electron is scattered in the *zx*-plane as shown in Fig. 1, we obtain the cross section written as

3 $$\frac{{d}^{4}\sigma }{d{{\boldsymbol{p}}}_{f}^{3}d\,\sin \,{\theta }_{y}}=\frac{{\alpha }^{2}{w}_{0}^{2}|{\boldsymbol{q}}|}{4\pi m{E}_{f}|(k-{q}_{z}){q}_{x}-{q}_{z}|{{\boldsymbol{p}}}_{T}||}{\bar{W}}_{if}{[G(|L|,p,{w}_{0}|{{\boldsymbol{p}}}_{T}+{{\boldsymbol{q}}}_{T}|)]}^{2},$$

4 $${\bar{W}}_{if}=\frac{1}{2}\{\frac{m{q}_{z}^{2}}{|{\boldsymbol{q}}|({p}_{f}\cdot q)}+\frac{mk}{{({p}_{f}\cdot q)}^{2}}[|{{\boldsymbol{p}}}_{f}{|}^{2}-\frac{{({{\boldsymbol{p}}}_{f}\cdot {\boldsymbol{q}})}^{2}}{|{\boldsymbol{q}}{|}^{2}}]+\frac{{E}_{f}|{\boldsymbol{q}}|-{p}_{z}{q}_{z}}{m|{\boldsymbol{q}}|}+\frac{{p}_{z}}{({p}_{f}\cdot q)}[\frac{{q}_{z}({{\boldsymbol{p}}}_{f}\cdot {\boldsymbol{q}})}{|{\boldsymbol{q}}{|}^{2}}-{p}_{z}]\},$$

with |***q***| = *k* + *m* − *E*_*f*_, where *m* is the rest mass of the electron, *p*_*f*_ = (*E*_*f*_, ***p***_*f*_) = (*E*_*f*_, ***p***_*T*_, *p*_*z*_) and *q* ≡ (|***q***|, ***q***) = (|***q***|, ***q***_*T*_, *q*_*z*_) are the momenta of the final electron and the final photon, respectively. Note that this cross-section is independent of the initial photon helicity.

To calculate quantitatively the cross sections under various conditions, we take the incident photon energy, *k*, as 500 keV, at which Compton scattering dominates. Note that this energy is much higher than the kinetic energies of electrons in atoms and hence the motion of the electrons can be ignored. One of features of the LG wave function is the fact that it has a waist, where the size of the wave has the minimum value, and the wave size increases as the photon propagates beyond the waist as described in Eq. (1). This spread of the LG wave, which is determined by the photon energy *k* and the waist radius *w*_0_ [see Eqs (1, 2)], affects its Compton scattering cross section. Although *w*_0_ is a free parameter in the present calculation, it depends on the generation mechanism of *γ*-ray vortices. We consider the *γ*-ray vortices will be generated by fundamental processes such as the non-linear inverse Compton scattering^25^ and the high-order harmonic radiation from helical undulators^14,15^, where a single electron radiates a single photon. In such cases, it is expected that the waist radius correlates with the wave length of the generated photon. However, to our knowledge, there is no theoretical prediction in the framework of the quantum mechanics. Thus, we take *w*_0_ to be 25 pm that is approximately ten times of the wave length of the present incident photon (2.48 pm) for the following calculation. Note that, in this condition, the ratio of the spread of the LG wave to its length in momentum space, |***p***_*T*_ + ***q***_*T*_|/*k*, is approximately 0.032 to satisfy the paraxial approximation for the LG wave function. The proper description of Compton scattering for narrow waists requires, in general, the incorporation of local polarization effects, but this effect is negligibly small for the present condition of |***p***_*T*_ + ***q***_*T*_|/*k* = 0.032.

**Figure 2:**
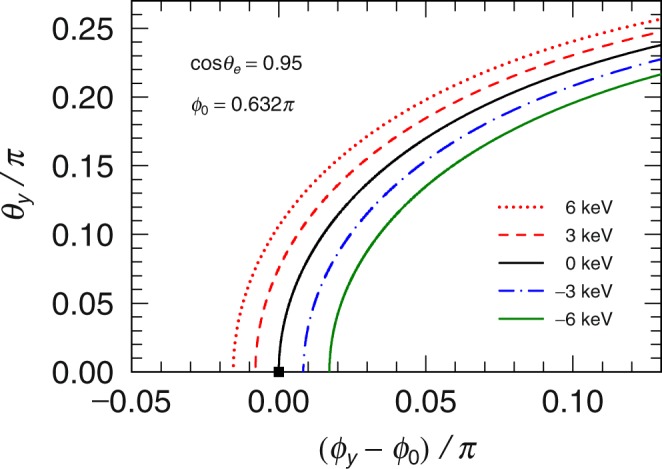
The directions of the scattered photons at the fixed momenta of the final electrons, when *cosθ*_*e*_ = 0.95. *ϕ*_0_ means the scattered angle in the case of standard Compton scattering, where the photon is scattered at the angle presented by the filled square. The dotted, dashed, solid, dot-dashed, and dpt-dot-dashed lines represent the results when the energy difference from that in standard Compton scattering are Δ*E* = 6 keV, 3 keV, 0 keV, −3 keV, and −6 keV, respectively.

We discuss the calculated cross section compared with that of standard Compton scattering with plane wave photons. In the case of the standard Compton scattering, the photon is scattered in the *zx*-plane (*θ*_*y*_ = 0) and the scattered angle of the photon, *ϕ*_0_, around the *y*-axis is uniquely determined when the scattered angle of the electron is determined because of the conservation law of momentum. In contrast, for the LG wave photon, the final photon momentum distributes, satisfying the relation *p*_*z*_ + *q*_*z*_ + (***p***_*T*_ + ***q***_*T*_)^2^/2*k* − *k* = 0 as shown in Fig. 2. This result exhibits that the strengths of the cross-sections distribute out of the *zx*-plane. In addition, the energy of the scattered photon can also be shifted from that of the standard Compton scattering. Figure 2 shows the distribution of the differential cross sections for various Δ*E*, which is the energy difference between the energy of the scattered photon with the LG wave and that with the plane wave. This energy shift, Δ*E*, correlates with the angular shift from the *zx*-plane, *θ*_*y*_/*π*. Note that these shifts have been known in the previous non-relativistic calculation^6^.

**Figure 3:**
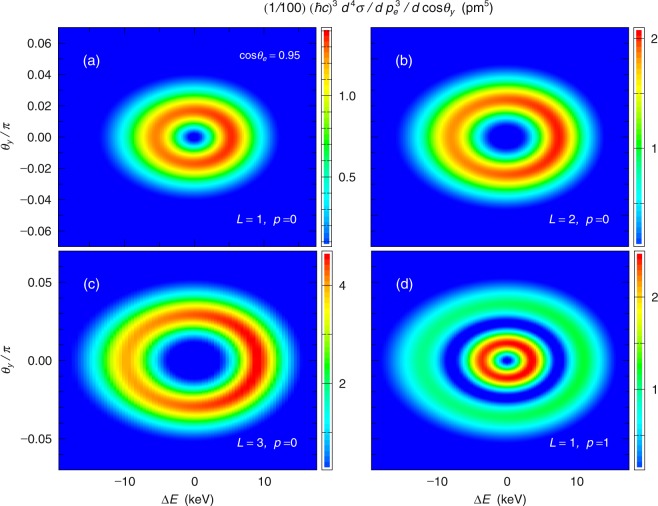
The contour plots of the differential cross-section of Compton scattering integrated over the azimuthal angle *ϕ*_*y*_ at *cosθ*_*e*_ = 0.95 when *L* = 1, *p* = 0 (**a**), *L* = 2, *p* = 0 (**b**), *L* = 3, *p* = 0 (**c**), and *L* = 1, *p* = 1 (**d**). The horizontal axis shows the energy difference Δ*E* from that in standard Compton scattering, and the vertical axis shows the polar angle between *zx*-plane and the scattered photon angle.

To present clearly the relationship between energy and angular shifts, we show the contour plots of the differential cross sections in Fig. 3, where the horizontal axis shows the energy difference Δ*E* and the vertical axis shows the polar angle along the *y*-axis. The strengths are exactly zero at *θ*_*y*_ = 0 and Δ*E* = 0, at which the strength appears in the standard Compton scattering. This result originates from the fact that the amplitude of the LG wave function is zero along the photon propagation axis when the projection of the orbital angular momentum of the incident LG photon, *L*, is not zero. The panels in Fig. 3 show the annulus structures, which reflect the distribution in the amplitude of the incident photon with the LG wave function. The momentum of the LG photon distributes out of the propagation axis to form the annulus. The size of the annulus increases with increasing *L* in the case of *p* = 0. This trend is also observed for *L* > 3. These results correspond to the shape of the photon wave function represented by the Laguerre function ${{\mathscr{L}}}_{p}^{|L|}$. One of other features of this function is that it has a node/nodes in the transverse direction for *p* > 0. As the node number, *p*, increases, the number of annuluses increases as shown in Fig. 3(b). These results indicate that when the energy of the scattered photon and both the momenta of the scattered photon and electrons are simultaneously measured, the amplitude distribution of the incident LG wave photon can be obtained.

**Figure 4:**
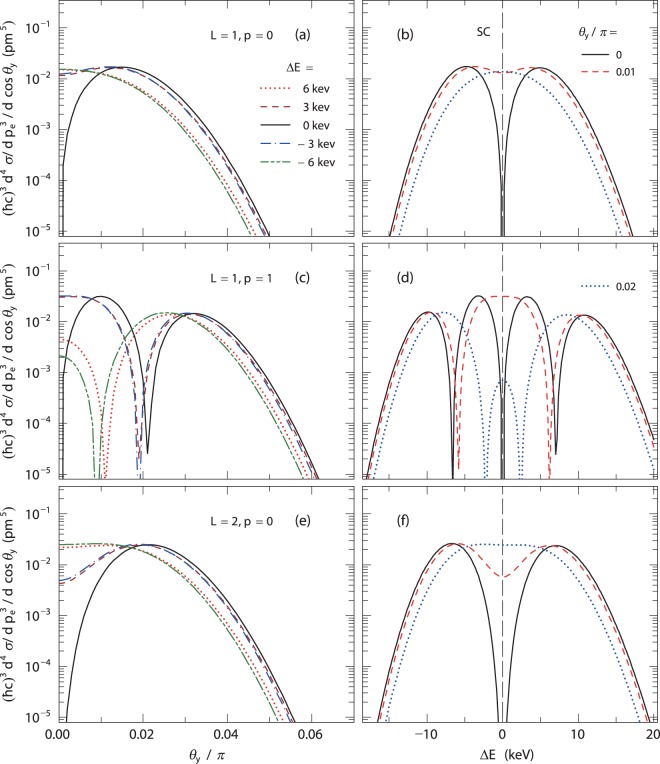
The differential cross-section of Compton scattering when *cosθ*_*e*_ = 0.95. The left panels show the *θ*_*y*_ dependence when *L* = 1, *p* = 0 (**a**), *L* = 1, *p* = 1 (**c**), and *L* = 2, *p* = 0 (**e**). The dotted, dashed, solid, dot-dashed, and dot-dot-dashed lines represent the results when Δ*E* = 6 keV, 3 keV, 0 keV, −3 keV, and −6 keV, respectively. The right panels show their energy spectra of the scattered photons, when *L* = 1, *p* = 0 (**b**), *L* = 1, *p* = 1 (**d**), and *L* = 2, *p* = 0 (**f**). The solid, dashed, and dotted lines represent the results when *θ*_*y*_/*π* = 0, 0.01, and 0.02, respectively. The long dashed lines indicate the results in the standard Compton (SC) scattering.

Let us discuss the result quantitatively. We show the differential cross sections at cos*θ*_*e*_ = 0.95 when *L* = 1 and *p* = 0 (a, b), *L* = 1 and *p* = 1 (c, d) and *L* = 2 and *p* = 0 (e, f) in Fig. 4. The left panels (a, c, e) present the angular dependence of the scattered photons. It is again confirmed the result that the strengths are exactly zero at *θ*_*y*_ = 0 and Δ*E* = 0 (see the solid lines), at which the strength appears in the standard Compton scattering. In contrast, for Δ*E* ≠ 0, the cross sections have non-zero values at *θ*_*y*_ = 0. The position of the peak depends on *L*. In the case of *L* = 1 (a), the peak with Δ*E* = 0 is located at *θ*_*y*_/*π* = 0.015. For *L* = 2 (e), the peak position is *θ*_*y*_/*π* = 0.021. As *L* increases, the peak position shifts toward a larger polar angle, which corresponds to the shape of the photon wave function represented by the Laguerre function ${ {\mathcal L} }_{p}^{|L|}$. This trend is consistent with the correlation between the size of the annulus and *L* in Fig. 3. Furthermore, the number of the peaks correlates with the node number, *p*. In the case of (c), there are two peaks, which originate from the fact that the photon wave function has a node in the transverse direction for *p* = 1. The right panels (b, d, f) present the expected energy spectra of the scattered photons. In the case of the standard Compton scattering, the energy is uniquely determined (see long dashed-lines); however, the energy of *γ*-ray vortices spreads. In all cases of *θ*_*y*_ = 0, the cross sections at Δ*E* = 0 are zero (see the solid lines), whereas for *θ*_*y*_ ≠ 0 the cross sections at Δ*E* = 0 have non-zero values.

**Figure 5:**
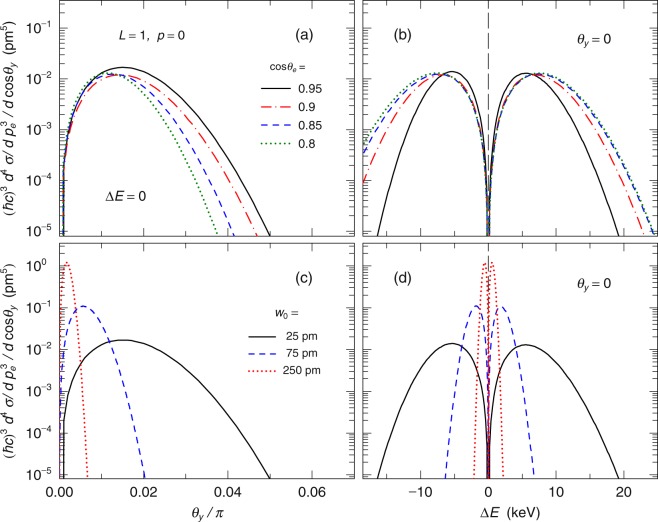
Figures show the *θ*_*y*_ dependence and its energy spectra of the scattered photons, when *L* = 1, *p* = 0. The long dashed lines in (**b**,**d**) indicate the results in the standard Compton (SC) scattering. The panels (a and b) show the *θ*_*y*_ dependence and its energy spectra of the scattered photons for various scattered electron angles. The panels (c and d) show those for various waist radius *w*_0_. The solid, dashed, and dotted lines represent the results when *w*_0_ = 25 pm, 75 pm, and 250 pm, respectively.

These angular and energy shifts depend on the scattered electron angle. Figure 5(a,b) show the differential cross sections and energy spectra for various scattered electron angles. As the scattered electron angle, *θ*_*e*_, increases, the energy distribution becomes broader [see Fig. 5(a)]. This suggests that a measurement at a large electron scattered angle of *cosθ*_*e*_ < 0.95 is easier than that at *cosθ*_*e*_ = 0.95. In contrast, the *θ*_*y*_ distribution becomes narrower with increasing *θ*_*e*_; however, at the large angle such as *cosθ*_*e*_ = 0.8, the *θ*_*y*_ distribution is still broad to measure the energy distribution [see Fig. 5(b)]. These results show the present proposed method does not depend strongly on the electron scattered angle.

In the present calculation, we have assumed the waist radius *w*_0_ as 25 pm. Because the energy and angular shifts also depend on the waist radius, we calculate their *w*_0_ dependence [see Fig. 5(c,d)]. With increasing *w*_0_, the spread of the LG wave decreases and thus both the shifts decrease. However, even if *w*_0_ is 250 pm, which is approximately 100 times of the wave length of the incident photon, the energy shift is as large as approximately 1 keV, which could be measured by a typical semi-conductor detector. In contrast, when *w*_0_ is smaller than the present value, these shifts are larger than the present results. Note again that *w*_0_ depends on generation mechanism, which is expected to be calculated in quantum mechanics, but it is beyond the scope of the present study.

## Discussion

In a previous study^6^, Compton scattering of vortex light beams was calculated within a non-relativistic framework using first-order perturbation theory. It was shown that the angular distribution and the polarization of the scattered photons depend on the orbital angular momentum and the opening angle of the incident vortex beam^6^. It was also presented that cross sections for incident vortex beams with *L* ≥ 3 vanish because of the electric dipole approximation. As predicted by the previous study^6^, the present relativistic calculation shows that Compton scattering for *L* ≥ 3 has non-zero cross section [see Fig. 3(d)]. In addition, the present calculation gives the exact cross section considering the recoil effect of the electron for high energy *γ*-rays. The relativistic calculation has another advantage that it is possible to calculate with the spin alignment of the electron.

The initial distribution of the electron momentum broadens the distribution of the scattered photon, so-called “Doppler broadening”. This effect depends on the atomic number of a target material and is small for alkaline and alkaline earth metals^28^. When magnesium or calcium is used as a target, the angular distribution of the scattered photon for standard Compton scattering is as small as approximately 0.009 radian in Full Width at Half Maximum (FWHM). The Doppler broadening effect also depends on the energy of an incident *γ*-ray. When the energy of *γ*-rays is higher than 1 MeV, the effect becomes negligibly small^28^. Another method to reduce the Doppler broadening effect is the use of Compton scattering on free electrons with an energy lower than 1 keV, which could be provided from a low-energy high-current electron gun (for example, see ref.^29^).

As discussed previously, the energy and angular shifts depends on the waist radius *w*_0_. In the present assumed conditions of *w*_0_ = 25 pm, 75 pm, and 250 pm, the ratio of the energy shift to the expected energy at *θ*_*y*_ = 0 is in the range of ΔE/E = 0.001–0.01 [see Fig. 5(d)]. These energy shifts could be measured by semi-conductor detectors because their typical energy resolutions are approximately 0.001–0.002 in FWHM. The measurement of the scattered angle of the photon, in general, is difficult rather than that of the scattered electron. When a collimator is used to restrict the detection angle of the scattered photon, the angular resolution of the scattered photon depends on a diameter of a hole in the collimator and a distance between the target and the collimator. If one locates a detector with a 0.5-mm diameter collimator 5-m downstream from a target, the angular resolution is in the range of *θ*_*y*_/*π* ∼ 0.001 and thus one could measure the angular shift of approximately *θ*_*y*_/*π* ∼ 0.002–0.02 in the cases of *w*_0_ = 25 pm, 75 pm, and 250 pm [see Fig. 5(c)]. Thus, it is possible to measure simultaneously both energy and angular shifts for each Compton scattering using standard experimental technique under the present assumed conditions.

In near future, the *γ*-ray vortex is expected to be generated in the laboratory. Optical devices to focus X-rays have been developed and thereby it is possible to focus 20-keV X-rays on a small area with a diameter of 7 nm^30^, in which the ratio of the diameter to its wave length is approximately 100. Beside focusing with optical devices, we consider the *γ*-ray vortices will be directly generated by fundamental processes such as the non-linear inverse Compton scattering with highly intense circularly polarized laser^25^ or the high-order harmonic radiation from helical undulators^14,15^, where a single electron is expected to radiate a single photon vortex under specific conditions. Taira *et al*.^25^ pointed out that *γ*-ray vortex generation by the non-linear inverse Compton scattering was probably demonstrated by another previous experiment^31^. The energy of the *γ*-rays generated by inverse Compton scattering is proportional to the energy of the incident photon and the square of the electron energy. The X-ray generation using a helical undulator has also been demonstrated^15^. Because the energy of the generated photon increases by increasing the electron energy, several hundred keV energy photons can be generated by the interaction with several GeV energy electrons. As stated in the introduction, it is also expected the generation by inverse Compton scattering with relatively low energy vortex photons on relativistic electrons^22–24^.

Thus, one can use *γ*-ray vortices carrying the total angular momentum higher than or equal to 2ℏ as tools to investigate nuclear and particle physics in the near future. Because the spin and parity of states excited by photon-induced reactions on nuclei are limited by the conservation laws of angular momentum, *γ*-ray vortices change possible excitation modes in photon-induced reactions^20,25^. For example, giant dipole resonances on J^*π*^ = 0^+^ nuclei are forbidden^25^. Photodisintegration reactions on deuteron with *γ*-ray vortices have been calculated^20^. Furthermore, high energy photon vortices also show new interactions in high energy physics^5^. Thus, when *γ*-ray vortices are available in the laboratory, they open a new frontier in nuclear and particle physics.

In summary, the present results indicate that, with the coincidence measurement of the scattered photon and electron, one can identify the angular momentum and the node number of the LG wave function for the incident photon. The correlation between the energy shift from that for standard Compton scattering and the angualr shift from *zx*-plane reflects the amplitude distribution of the incident wave function. Thus, the present proposed method is useful for the study of the nature of LG wave photons in addition to the verification of its generation.

## Method

We consider Compton scattering with a LG wave function photon at the energy *k* propagating along the z-direction on a rest electron (see Fig. 1). We also assume that the electron is scattered in the *zx*-plane and that the final photon wave function is the plane wave. The amplitude of Compton scattering in relativistic quantum mechanics^32^ is given by

5 $${S}_{if}={e}^{2}\int {d}^{4}x{d}^{4}y\,\overline{{\psi }_{f}}(x){\gamma }_{\mu }{S}_{F}(x,y){\gamma }_{\nu }{\psi }_{i}(y)[{A}_{f}^{\mu \ast }(x){A}_{i}^{\nu }(y)+{A}_{i}^{\mu }(x){A}_{f}^{\nu \ast }(y)],$$

where *e* is the elementary charge, *ψ*_*i*_ and *ψ*_*f*_ are the initial and final electron wave functions, respectively, *S*_*F*_ is the electron propagator, and ${A}_{i}\equiv ({A}_{i}^{0},{{\bf{A}}}_{i})$ and ${A}_{f}\equiv ({A}_{f}^{0},{{\bf{A}}}_{f})$ are the initial and final photon fields, respectively. We choose the Lorentz gauge and *A*_0_ = 0 for the photon field and define the final photon momentum as *q* ≡ (|***q***|, ***q***) = (|***q***|, ***q***_*T*_, *q*_*z*_). The electron and photon fields are written as

6 $$\psi (x)=\frac{1}{\sqrt{{\rm{\Omega }}}}U({\boldsymbol{p}},s){e}^{i{\bf{pr}}-i{E}_{p}t},\,\,{{\boldsymbol{A}}}_{i}({\boldsymbol{r}})=\frac{{\epsilon }_{i}({h}_{i})}{\sqrt{2k}}u({\boldsymbol{r}}){e}^{-ikt},\,\,{{\boldsymbol{A}}}_{f}({\boldsymbol{r}})=\frac{{\epsilon }_{f}({h}_{f})}{\sqrt{\mathrm{2|}{\boldsymbol{q}}|{\rm{\Omega }}}}{e}^{i{\bf{qr}}-i|q|t},$$

where Ω is the volume of the system, *U*(***p***, *s*) is the Dirac spinor of an electron with the momentum *p* = (*E*_*p*_, ***p***) and the spin *s*, *k* is the energy of the initial photon, *h*_*i*(*f*)_ indicates the helicity of the initial (final) photon, and the polarization vector satisfies *ϵ*_*f*_ ⋅ ***q*** = 0 and *ϵ*_*f*_ ⋅ *ϵ*_*f*_ = 1. We write the initial and final momenta of the electron as *p*_*i*_ = (*E*_*i*_, ***p***_*i*_) and *p*_*f*_ = (*E*_*f*_, ***p***_*f*_) = (*E*_*f*_, ***p***_*T*_, *p*_*z*_), respectively. The scattering amplitude is rewritten as

7 $$\begin{array}{rcl}{S}_{if} & = & \tfrac{{e}^{2}}{2\sqrt{{k}_{i}^{0}|{\boldsymbol{q}}|{\rm{\Omega }}}}\overline{U}({{\boldsymbol{p}}}_{f},{s}_{f})[{\rlap{/}{\epsilon }}_{f}{S}_{F}({p}_{f}+q){\rlap{/}{\epsilon }}_{i}+{\rlap{/}{\epsilon }}_{i}{S}_{F}({p}_{i}-q){\rlap{/}{\epsilon }}_{f}]U({{\boldsymbol{p}}}_{i},{s}_{i})\\  &  & \times \,\tilde{u}({{\boldsymbol{p}}}_{f}+{\boldsymbol{q}}-{{\boldsymbol{p}}}_{i})(2\pi )\delta ({E}_{f}+|{\boldsymbol{q}}|-{E}_{i}-k)\end{array}$$

with *ϵ*_*i*,*f*_ = (0, *ϵ*_*i*,*f*_) and

8 $${S}_{F}(p)=\frac{\rlap{/}{p}+m}{{p}^{2}+{m}^{2}+i\delta },\,\tilde{u}({\boldsymbol{k}})=\int d{\boldsymbol{r}}{e}^{-ik\cdot r}u({\boldsymbol{r}}).$$

Then, the cross-section is given by

9 $$d\sigma =\frac{{e}^{4}}{4k{E}_{i}}{W}_{if}{|\tilde{u}({{\boldsymbol{p}}}_{f}+{\boldsymbol{q}}-{{\boldsymbol{p}}}_{i})|}^{2}(2\pi )\delta ({E}_{f}+|{\boldsymbol{q}}|-{E}_{i}-k)\frac{d{\boldsymbol{q}}}{{\mathrm{(2}\pi )}^{3}|{\boldsymbol{q}}|}\frac{d{{\boldsymbol{p}}}_{f}}{{\mathrm{(2}\pi )}^{3}{E}_{f}}$$

with

10 $${W}_{if}={E}_{i}{E}_{f}{|\overline{U}({{\boldsymbol{p}}}_{f},{s}_{f})[{\rlap{/}{\epsilon }}_{f}{S}_{F}({p}_{f}+q){\rlap{/}{\epsilon }}_{i}+{\rlap{/}{\epsilon }}_{i}{S}_{F}({p}_{i}-q){\rlap{/}{\epsilon }}_{f}]U({{\boldsymbol{p}}}_{i},{s}_{i})|}^{2}\mathrm{.}$$

We assume the initial rest electron, the initial photon parallel to the *z*-direction, and no observation of the final photon polarization. Then, we substitute *p*_*i*_ = (*m*, 0, 0, 0) and ${\epsilon }_{i}({h}_{i})=\mathrm{(1},i{h}_{i},\mathrm{0)/}\sqrt{2}$ with *h*_*i*_ = ±1. We average the spin of the initial electron and sum over the spin of the final electron and the polarization of the final photon. As the result, the cross-section is written as

11 $$d\sigma =\frac{{\alpha }^{2}{w}_{0}^{2}}{8{\pi }^{3}mk|{\boldsymbol{q}}|{E}_{f}}{\bar{W}}_{if}|\tilde{u}({{\boldsymbol{p}}}_{f}+{\boldsymbol{q}}{)|}^{2}\delta ({E}_{f}+|{\boldsymbol{q}}|-m-k)d{\boldsymbol{q}}d{{\boldsymbol{p}}}_{f},$$

with

12 $$\begin{array}{rcl}{\bar{W}}_{if} & = & \frac{1}{2}\sum _{{s}_{i},{s}_{f},{h}_{f}}{W}_{if}=\frac{1}{2}\{\frac{m{q}_{z}^{2}}{|{\boldsymbol{q}}|({p}_{f}\cdot q)}+\frac{mk}{{({p}_{f}\cdot q)}^{2}}[|\,{{\boldsymbol{p}}}_{f}{|}^{2}-\frac{{({{\boldsymbol{p}}}_{f}\cdot {\boldsymbol{q}})}^{2}}{|{\boldsymbol{q}}{|}^{2}}]\\  &  & +\frac{{E}_{f}|{\boldsymbol{q}}|-{p}_{z}{q}_{z}}{m|{\boldsymbol{q}}|}+\,\frac{{p}_{z}}{({p}_{f}\cdot q)}[\frac{{q}_{z}({{\boldsymbol{p}}}_{f}\cdot {\boldsymbol{q}})}{|{\boldsymbol{q}}{|}^{2}}-{p}_{z}]\},\end{array}$$

where *α* is the fine structure constant. Note that these cross-sections are independent of the initial photon helicity *h*_*i*_. The Fourier transformation of *u*(***r***) becomes

13 $$\tilde{u}({\boldsymbol{Q}})=\sqrt{\frac{{\mathrm{(2}\pi )}^{3}}{2{R}_{z}}}{e}^{i(p+|L\mathrm{|/2)}\pi }{e}^{iL{\varphi }_{q}}{w}_{0}G[|L|,p;\frac{{w}_{0}{Q}_{T}}{\sqrt{2}}]\delta ({Q}_{z}+\frac{{Q}_{T}^{2}}{2k}-k),$$

where the initial photon momentum is given by ***Q*** = ***p***_*f*_ + ***q*** with ${Q}_{T}\equiv \sqrt{{Q}_{x}^{2}+{Q}_{y}^{2}}$ and *ϕ*_*q*_ is the azimuthal angle of the momentum ***Q*** along the *z*-axis.

To illustrate the final photon momentum distribution, we take *y*-direction to be a new principal axis (see Fig. 1) and write the final photon momentum as ***q*** = |***q***|(*cosθ*_*y*_*sinϕ*_*y*_, *sinθ*_*y*_, *cosθ*_*y*_*cosϕ*_*y*_). Combining Eqs (11) and (13), we obtain the cross-section for the incident photon of the LG wave. We integrate the cross-section over |***q***| and *ϕ*_*y*_ for a fixed electron momentum to obtain the final differantial cross section.
